# “Malancha” [*Alternanthera philoxeroides* (Mart.) Griseb.]: A Potential Therapeutic Option against Viral Diseases

**DOI:** 10.3390/biom12040582

**Published:** 2022-04-14

**Authors:** Lutfun Nahar, Sushmita Nath, Satyajit D. Sarker

**Affiliations:** 1 Laboratory of Growth Regulators, Institute of Experimental Botany ASCR & Palacký University, Šlechtitelů 27, 78371 Olomouc, Czech Republic; 2 Centre for Natural Products Discovery (CNPD), School of Pharmacy and Biomolecular Sciences, Liverpool John Moores University, James Parsons Building, Byrom Street, Liverpool L3 3AF, UK; sushmitanath84@gmail.com

**Keywords:** *Alternanthera philoxeroides*, Amaranthaceae, antibacterial, anticancer, antiviral, bioactivities, biomolecular interactions, flavonoids, saponins, mechanisms

## Abstract

*Alternanthera philoxeroides* (Mart.) Griseb., commonly known as “Alligator weed” in English, and “Malancha” in Bengali, is a leafy vegetable from the family Amaranthaceae A. L. de Jussieu. This species is native to China, particularly to the provinces around the Yangtze River, other Far East and South-East Asian countries, and countries from other continents (e.g., South America). This plant also grows in certain areas in Australia, New Zealand, and the USA. While in Bangladesh the leaves of this plant are consumed as a vegetable, in China, this plant has been used widely as a traditional remedy for the treatment of various viral diseases (e.g., measles, influenza, and haemorrhagic fever). Flavonoids and saponins are the two largest groups of phytochemicals produced by this plant, and the antiviral property of this plant and its compounds has been studied extensively. This review article reviews all published literature on this plant and critically appraises its phytochemical profile linking to biomolecular interactions and therapeutic potential, particularly, against viral diseases.

## 1. Introduction

**Figure 1:**
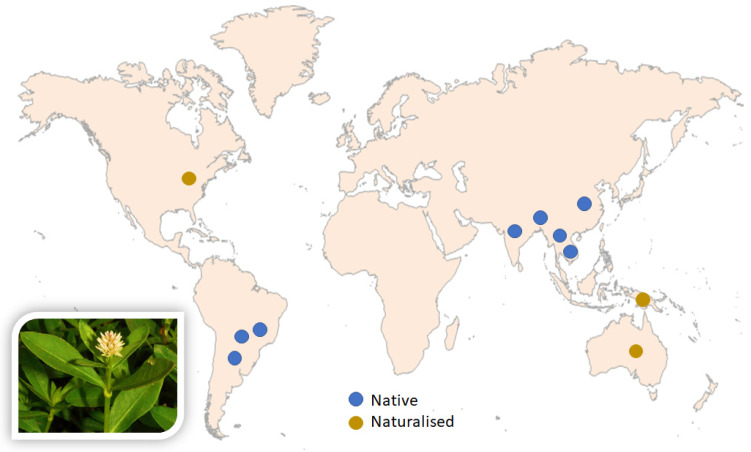
Distribution map of *Alternanthera philoxeroides*.

*Alternanthera philoxeroides* (Mart.) Griseb., commonly known as “Alligator weed” in English, “Malancha shak or Malancha” in Bengali, and “Shergitti” in Santal languages, is a leafy vegetable from the family Amaranthaceae A. L. de Jussieu [1,2,3]. This perennial polymorphic herb is native to China, particularly to the provinces around the Yangtze River, Far East, and South-East Asian countries such Bangladesh, India, Myanmar, and Thailand [1,4,5], and countries from other continents (e.g., Argentina, Brazil, and Paraguay [6,7] (Figure 1). This species also grows in certain areas in Australia, New Zealand, and the USA, where it is considered as an obnoxious invasive weed. In fact, it is one of the worst hazardous weeds that can rapidly invade both terrestrial and aquatic habitats.

While in Bangladesh the leaves of this plant are consumed as a vegetable, in China, this plant has been used extensively as a traditional remedy for various viral diseases (e.g., measles, influenza, and haemorrhagic fever) [4,5,8,9,10,11,12]. This plant has traditionally been used in India as a remedy for anaemia, for the treatment of diarrhoea and dysentery in Bangladesh, and to treat certain blood conditions, fever, post-natal depression, wounds and to stimulate milk secretion in Thailand [11,13].

Despite being known as a hazardous invasive weed, because of its traditional phytotherapeutic uses, this plant has been widely investigated to furnish the presence of several biologically active secondary metabolites, and to reveal various medicinal values and therapeutic potential, particularly, against viral infections. However, no review article is available to date that appraises the findings of those investigations. Therefore, this review article critically appraises the phytochemical profile linking to biomolecular interactions and the therapeutic potential of *A. philoxeroides* and its purified compounds based on the published literature available to date and also explores the true potential of this plant as an antiviral therapeutic option.

## 2. Phytochemical Profile and Biomolecular Interactions

Several phytochemical studies have been performed on this plant [4,5,6,7,8,9,10,11,12,13,14,15,16,17,18]. While some of these are merely preliminary qualitative and quantitative phytochemical screening without the isolation of any compounds, there are, however, several other thorough phytochemical studies that have led to the isolation and identification of various secondary metabolites from *A. philoxeroides*. Among at least 60 different isolated compounds, mainly from the aerial parts of this plant, flavonoids and saponins form the two major classes. The phytochemical profile as revealed by different phytochemical studies on *A. philoxeroides* is discussed in the following subsections under the headings of various phytochemical classes, and their interactions with various biomolecules/macromolecules are also briefly discussed.

### 2.1. Alkaloids

**Figure 2:**
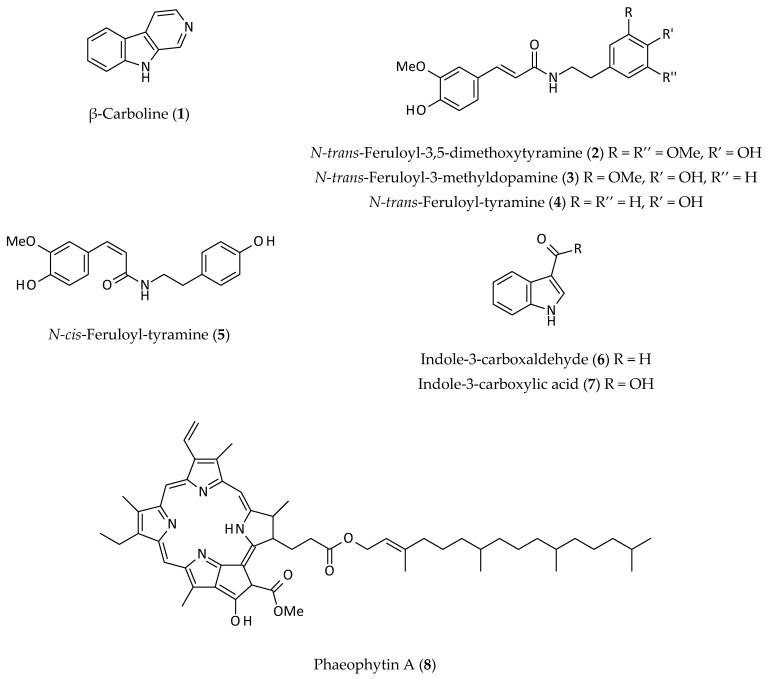

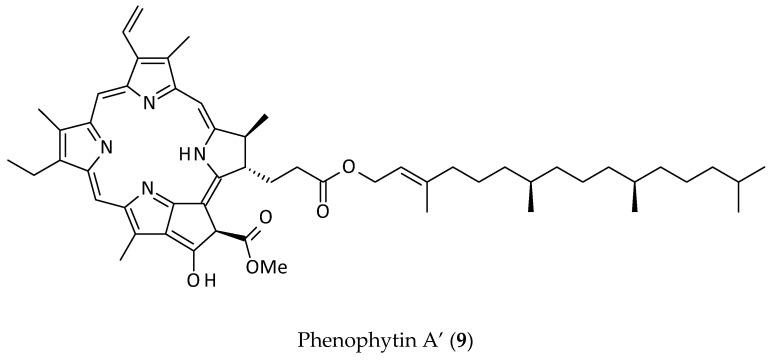
Alkaloids from *Alternanthera philoxeroides*.

At least nine alkaloids, belonging to the classes of β-carboline, indole, phaeophytin and tyramine alkaloids (**1**–**9**) (Figure 2), have been isolated from the aerial parts of *A. philoxeroides* [5,11,12,14,16]. The first two alkaloids, phaeophytin A (**8**) and phaeophytin A’ (**9**), were isolated from the aerial parts of this plant by Fang et al. [14]. Later, further alkaloids, *N-trans*-feruloyl-3,5-dimethoxytyramine (**2**), *N-trans*-feruloyl-3-methyldopamine (**3**), *N-trans*-feruloyl-tyramine (**4**), *N-cis*-feruloyl-tyramine (**5**), indole-3-carboxaldehyde (**6**) and indole-3-carboxylic acid (**7**) were reported from this plant in subsequent years [5,12]. Tyramine derivatives (**2**–**5**), particularly feruloyl, cinnamoyl, or coumaroyl derivatives, are widespread in the plant kingdom. *N-cis*-feruloyl-tyramine (**5**) could be an artefact formed during the isolation process as *cis*/*trans* isomerism is usually induced by light. However, natural existence of *cis*- and *trans*-isomers of various natural compounds is not uncommon. Tyramine and its derivatives have been shown to act on various biomolecular targets (e.g., monoamine oxidase (MAO), α and β1 adrenoreceptors), and thus to produce certain toxicological effects such as migraine and hypertension [19,20].

Phaeophytins or pheophytins (**8** and **9**) are chlorophyll molecules without a magnesium ion (Mg^2+^). They are essential parts of photosynthesis in plants, and act as the first electron carrier intermediate in the electron transfer pathway of the photosystem II pathway.

β-Carboline (**1**), reported from the aerial parts of *A. philoxeroides* [16], has an indole unit fused with a six membered heteroaromatic ring (pyridine ring), and is the basic structure of *ca.* 100 alkaloids with various pharmacological properties. This group of compounds are well distributed in prokaryotes, plants, and animals. Many β-carboline analogues can intercalate into DNA and can inhibit cyclin dependent kinase (CDK), topoisomerase, and MAO [21]. These alkaloids also interact with benzodiazepine and 5-hydroxy-serotonin receptors, and possess anticonvulsant, antimicrobial, antitumour, antiviral, antiparasitic, anxiolytic, hypnotic, and sedative properties [22].

### 2.2. Anthraquinones

**Figure 3:**
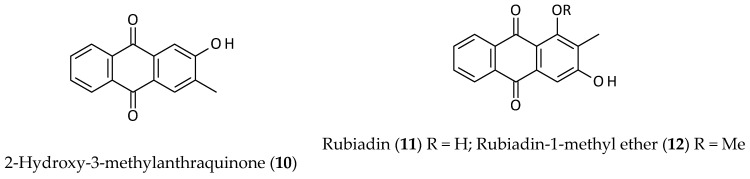
Anthraquinones from *Alternanthera philoxeroides*.

Anthraquinones, also known as anthracenedione or 9,10-dioxoanthracene, are tricyclic aromatic compounds based on the anthracene skeleton [23]. Anthraquinones form a large group of pharmacologically active phytochemicals found in various plants (e.g., *Aloe*, *Cascara, Cassia, Frangula,* and *Senna* species). In plants, they often occur in their glycosidic forms. Three anthraquinones, 2-hydroxy-3-methylanthraquinone (**10**), rubiadin (**11**), and rubiadin-1-methyl ether (**12**) (Figure 3), were isolated from an ethyl acetate (EtOAc) extract of the aerial parts of *A. philoxeroides* by repeated column chromatography on silica gel [12]. None of these anthraquinones is particularly unique to this plant and can be found in other plants. 2-Hydroxy-3-methylanthraquinone (**10**) is found in various other Chinese medical plants (e.g., it was isolated as an anticancer principle from *Hedyotis difffusa*) [24]. Similarly, bioactive rubiadin (**11**) and its derivatives are also found in other plants (e.g., *Morinda citrifolia* and *Rubia cordifolia*) [25,26]. It can be noted that rubiadin (**11**), which fits the Lipinski’s rule of five for drug-likeness properties, is known to possess antibacterial, anticancer, antidiabetic, antifungal, anti-inflammatory, antimalarial, antioxidant, antiviral, hepatoprotective, and neuroprotective properties [26]. Natural anthraquinones usually bind to serum albumin, DNA, and glutathione (GSH) [27,28].

### 2.3. Flavonoids

**Figure 4:**
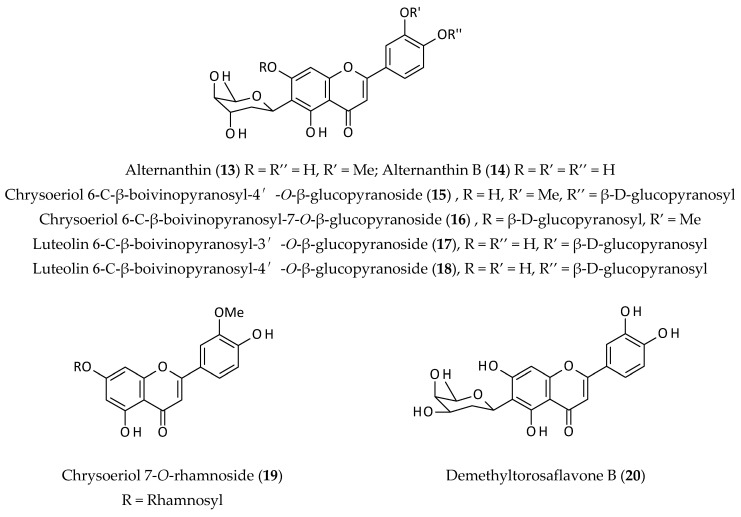

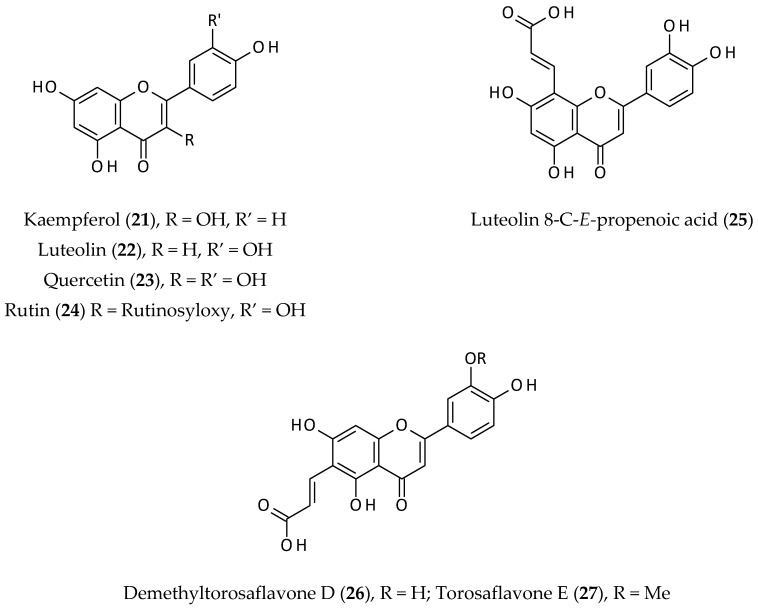
Flavonoids from *Alternanthera philoxeroides*.

Flavonoids, having a benzo-*γ*-pyrone skeleton, are one of the largest groups of phenolic/polyphenolic compounds produced by *A. philoxeroides* [4,5,6,10,11,12,29]. To date, at least 15 different flavonoids and their glycosides (**13**–**27**) have been reported from this plant (Figure 4).

Flavonoids of this plant mainly possess chrysoeriol (**19**) and luteolin (**22**) skeletons, and alternanthins appear to be the signature flavonoids of this species [4,5,6,10,11]. One of the unique features of some flavonoids of this plant is the presence of the deoxysugar boivinopyranose, which forms the C-glycosidic link to the flavonoid aglycone, as found in alternanthin (**13**), alternanthin B (**14**), and a few other chrysoeriol/luteolin glycosides (**15**–**18**).

While three propenoic acid substituted flavonoids, demethyltorosaflavone D (**26**), luteolin 8-C-*E*-propenoic acid (**25**), and torosaflavone (**27**), appear to be among the less common natural flavonoids, kaempferol (**21**), luteolin (**22**), quercetin (**23**), and rutin (**24**) are among the most abundant natural flavonoids. Alternanthin (**13**), the signature flavonoid of this plant, was first isolated as a novel flavonoid C-glycoside containing the rare deoxysugar boivinose from an ethanol (EtOH) (95%) extract of the stems and leaves of this plant using repeated column chromatography on silica gel and the structure was deduced with the help of UV–Vis and nuclear magnetic resonance (NMR) experiments, particularly a series of 1D nOe spectral analyses [4]. Khamphukdee et al. [11] used solvent partitioning (with chloroform and EtOAc) of an EtOH extract of the aerial parts of this plant, followed by column chromatography on silica gel to isolate demethyltorosaflavone B (**20**) and torosaflavone (**27**) together with five other previously reported flavonoids. Among the 15 isolated flavonoids from *A. philoxeroides*, 11 are flavone (no substitution at C-3) and the remaining four are flavonol derivatives (oxygenation at C-3).

Flavonoids possess various pharmacological properties including anti-ageing, anticancer, anti-inflammatory, antioxidant, antimicrobial, antiparasitic, antiviral, cancer chemopreventive, cardioprotective, hepatoprotective, and immunomodulatory properties [23]. Several flavonoids (e.g., quercetin (**23**) and rutin (**24**)), are used in various traditional medicinal formulations and in some ‘over the counter’ medications [23,30,31,32]). In addition to medicinal values, natural flavonoids are also important as dietary components possessing health-promoting properties due to their high antioxidant capacity [23]. Flavonoids, depending on their structural features, can bind to several biomolecules including various enzymes. The binding affinity can be quite variable because of the structural diversity that flavonoids possess [30,33,34,35]. For example, Liu et al. [35] demonstrated differential binding affinities of some known flavonoids with bovine serum albumin, and the order of affinities was hesperetin (K_A_ = 5.59 × 10^5^) > quercetin (4.94 × 10^5^) > naringenin (3.04 × 10^5^) > isoquercitrin (4.66 × 10^4^) > icariin (3.60 × 10^4^) > rutin (1.65 × 10^4^) > hesperidin (2.50 × 10^3^) > naringin (8.70 × 10^2^). The differences in the rates of binding of these flavonoids to serum albumin were assumed to be due to differences in hydrophobicity, functional groups, steric hindrance, and the spatial arrangements of substituents.

The antiviral property of certain flavonoids is associated with their inhibiting activity against enzymes such as human immune deficiency virus (HIV-1) reverse transcriptase, proteinase, protein kinases, and DNA-polymerases [30,36]. For example, flavonoids baicalein, robustaflavone, and hinokiflavone inhibit HIV-1 reverse transcriptase. The interactions of flavonoids with macromolecules (e.g., lipoproteins, proteins, chromatin, DNA, and cell-signalling molecules in human diseases have been well documented) [37,38].

### 2.4. Megastigmanes

**Figure 5:**
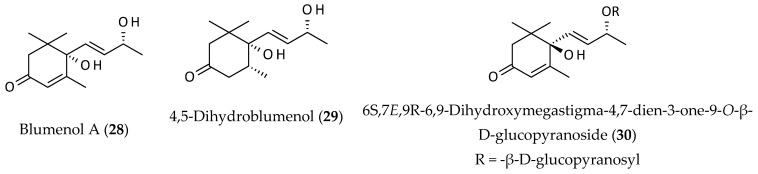
Megastigmanes from *Alternanthera philoxeroides*.

Megastigmanes are a large group of 13 carbon atoms containing nonterpenoidal natural products that are usually responsible for the characteristic aroma of certain fruits and plants [23]. Usually, these compounds possess a cyclohexane ring (often cyclohexanone or cyclohexen-one), a butyl side chain, and three other methyl groups. Fan et al. [12] isolated blumenol A (**28**) from an EtOH extract of the aerial parts of *A. philoxeroides,* while 4,5-dihydroblumenol (**29**) and 6S,7*E*,9R-6,9-dihydroxymegastigma-4,7-dien-3-one-9-*O*-β-D-glucopyranoside (**30**) with antitumour properties were reported from an *n*-butanol extract of this plant [9] (Figure 5).

Natural megastigmanes are known to have various bioactivities including anticancer, antimicrobial, antioxidant, and antiviral properties [39,40]. For example, blumenol A (**28**) is weakly cytotoxic to human cancer/tumour cells [39], whereas three megastigmane glucosides isolated from *Lyonia ovalifolia* have been shown to possess antiviral properties against the Coxsackie B3 virus [40]. A similar antiviral property was also observed with two megastigmane glycosides isolated from *Pinus densiflora* against the human influenza A virus [41]. Certain megastigmanes have been shown to bind to various proteins and enzymes including nucleotide-binding oligomerisation-domain protein and other macromolecules to exert pharmacological properties such as anti-inflammatory, anticancer and antiviral properties [42].

### 2.5. Other Phenolics

**Figure 6:**
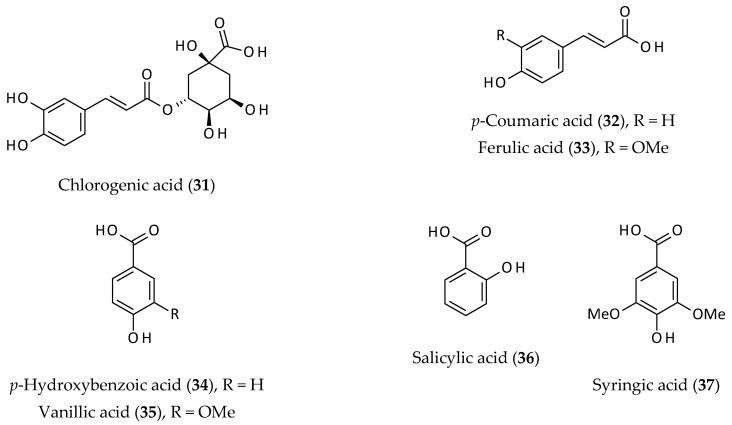
Other phenolics from *Alternanthera philoxeroides*.

Simple phenolics (**31**–**37**) and coumarins have been reported from the aerial parts of *A. philoxeroides* [6,11,12,43,44]. However, none of the coumarins or coumarin analogues were isolated and identified from this plant, but just reported based on preliminary phytochemical screening [43,44]. Chlorogenic acid (**31**), *p*-coumaric acid (**32**), ferulic acid (**33**), *p*-hydroxybenzoic acid (**34**), salicylic acid (**36**), syringic acid (**37**), and vanillic acid (**35**) (Figure 6) are among the simple phenolics (apart from flavonoids) isolated from the aerial parts and/or leaves of this plant [6,11,12]. All these phenolic compounds are known to possess various biological activities (e.g., analgesic, anti-inflammatory, antioxidant, anticancer, antimicrobial, and antitumour properties), and are widely distributed in the plant kingdom [23].

Binding ability of simple phenolic compounds (e.g., chlorogenic acid (**31**), ferulic acid (**33**) and gallic acid), to macromolecules (e.g., proteins including human serum albumin, bovine serum albumin, soy glycinin, and lysozyme is well-known) [45,46,47]. A vast majority of phenol–protein binding takes place through covalent and non-covalent (hydrogen, hydrophobic and ionic bonds) binding, the former being an irreversible process [47]. However, covalent and non-covalent binding such as between chlorogenic acid (**31**) and proteins occurs simultaneously. The interactions between various natural phenolic acids and plasma proteins, especially serum albumin, have been studied quite extensively [47]. Simple phenolics such as ferulic acid (**33**) and rosmarinic acid could inhibit amyloid β protein aggregation, which is an important feature of Alzheimer’s disease, and thus, food rich in phenolics could potentially reduce the incidence of Alzheimer’s disease [48].

### 2.6. Saponins

**Figure 7:**
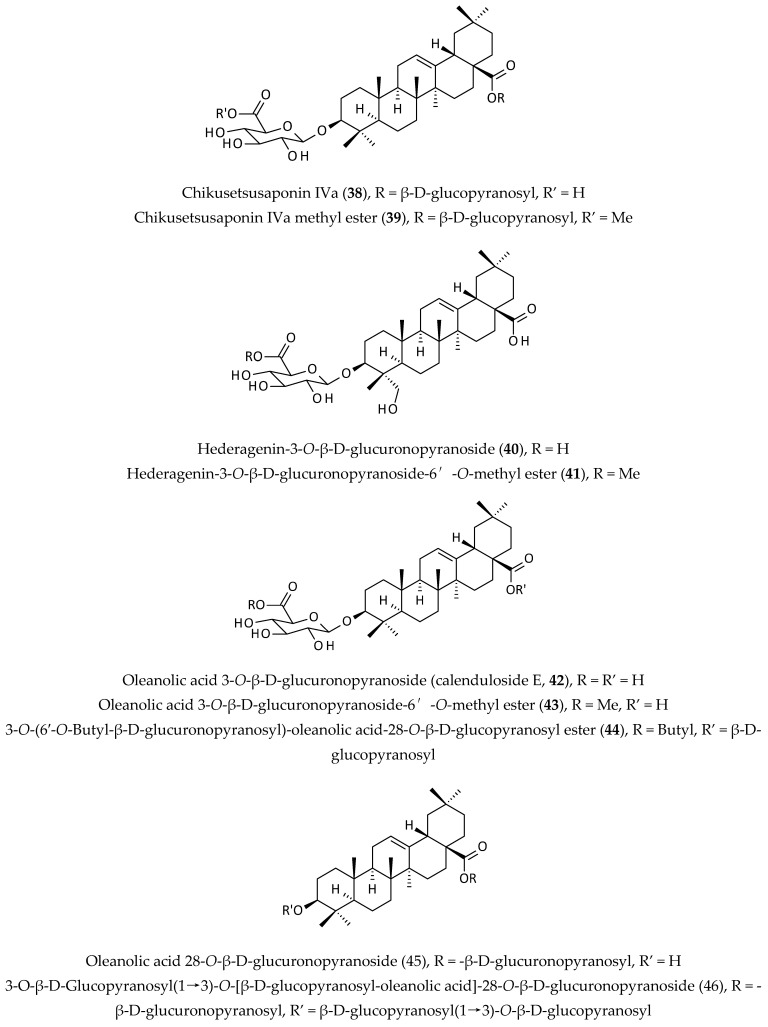

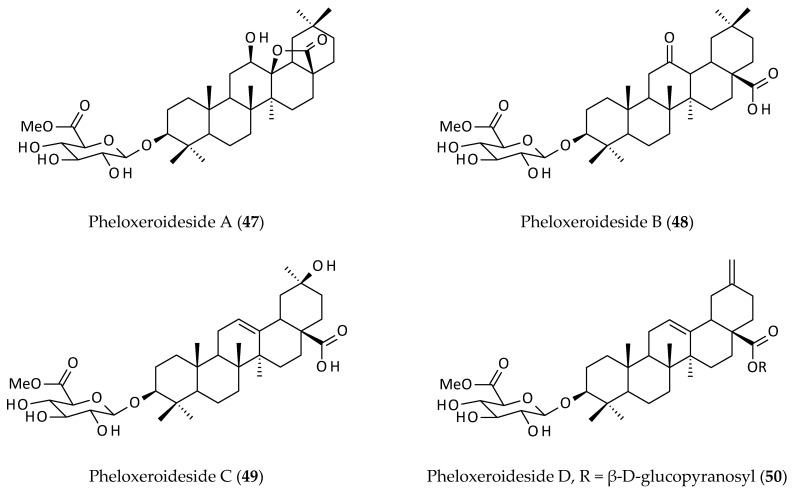
Saponins from *Alternanthera philoxeroides*.

Saponins are ‘foam-forming’ natural products comprising a triterpene or steroidal aglycone unit and multiple sugar units, often in the form of di-, tri-, tetra-saccharides, and have various pharmacological and toxicological properties [23,49]. This is the second largest group of phytochemicals produced by *A. philoxeroides*, and to date, at least 13 different saponins (**38**–**50**) (Figure 7) have been isolated from the aerial parts of this plant [8,9,50,51,52].

Dogra and Ojha [50] were the first to report on the presence of saponins in this plant, which was followed by the studies undertaken by a few other groups resulting in the isolation of calenduloside E (**42**), chikusetsusaponin IVa (**38**), chikusetsusaponin IVa methyl ester (**39**), hederagenin 3-*O*-β-D-glucuronopyranoside (**40**), hederagenin 3-*O*-β-D-glucuronopyranoside-6′-*O*-methyl ester (**41**), oleanolic acid 3-*O*-β-D-glucuronopyranoside-6′-*O*-methyl ester (**43**), 3-*O*-(6′-*O*-butyl-β-D-glucuronopyranosyl)-oleanolic acid-28-*O*-β-D-glucopyranosyl ester (**44**), oleanolic acid 28-*O*-β-D-glucuronopyranoside (**45**), 3-*O*-β-D-glucopyranosyl(1→3)-*O*-[β-D-glucopyranosyl-oleanolic acid]-28-*O*-β-D-glucuronopyranoside (**46**), and pheloxeroidesides A-D (**47**–**50**) from various EtOH (95%) extracts of this plant [8,9,13,51,52].

In most cases, a combination of solvent partitioning of the resuspended dried EtOH extract in water with *n*-butanol and EtOAc, repeated column chromatography on silica gel, and reversed-phase preparative HPLC afforded isolation, and an extensive 1D and 2D NMR analyses together with mass spectrometric (MS) data analyses were required to isolate and elucidate the complex structures of those saponins [8,9,52].

Saponins are usually responsible for various pharmacological properties of medicinal plants as well as toxicities in some cases. Most notable pharmacological properties of saponins include anticancer, anticoagulant, anti-inflammatory, antimicrobial, hepatoprotective, hypocholesterolaemic, hypoglycaemic, immunomodulatory, and neuroprotective activities [49]. A few decades ago, possible interaction between natural saponins (quillaja saponins) and proteins (casein and soy proteins) and their influence on blood lipids, particularly low-density lipoprotein (LDL) cholesterol, was established [53]. Recently, it has been shown that *Ginseng* saponins, ginsenosides, bind to plasma lipid membranes to exert their pharmacological actions through the modulation of essential membrane proteins and the reorganisation of lipid bilayers [54]. As ginsenosides suppress cell proliferation, induce apoptosis, and inhibit efflux pumps, these saponins could be considered as candidates for anticancer and antimicrobial drug development [55].

### 2.7. Sterols

**Figure 8:**
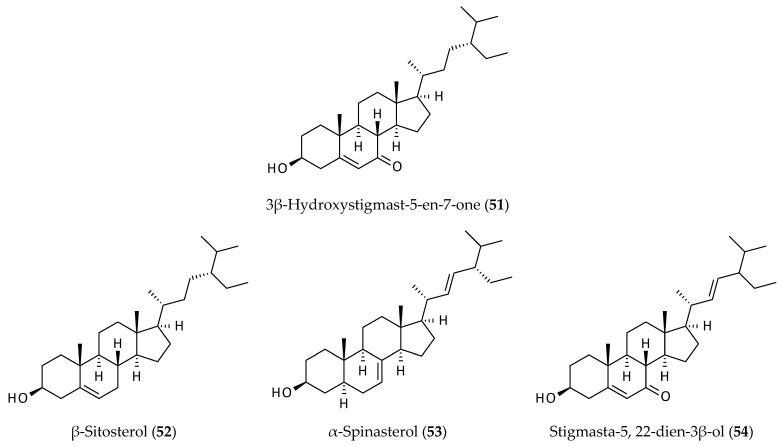
Steroids from *Alternanthera philoxeroides*.

Four well-known plant sterols, 3β-hydroxystigmast-5-en-7-one (**51**), β-sitosterol (**52**), α-spinasterol (**53**), and stigmasta-5, 22-dien-3β-ol (**54**), were isolated from the aerial parts of *A. philoxeroides* [12,14]) (Figure 8). These sterols are widely distributed in the plant kingdom but possess various biological properties and have health-protecting values [56,57,58,59]. One of the most prominent health effects of plant sterols is their ability to lower cholesterol level; long-term intake of certain plant sterols can lower serum cholesterol level to the extent expected to reduce clinical manifestation of coronary heart disease by over 20% without any detectable side effects [56]. Plant sterols interfere with sterol regulatory element-binding protein 2 and liver X receptor regulatory pathways resulting in a reduction in serum LDL cholesterol level and can be used as a therapeutic option for the management of blood cholesterol and atherosclerotic risks [60].

### 2.8. Terpenoids

**Figure 9:**
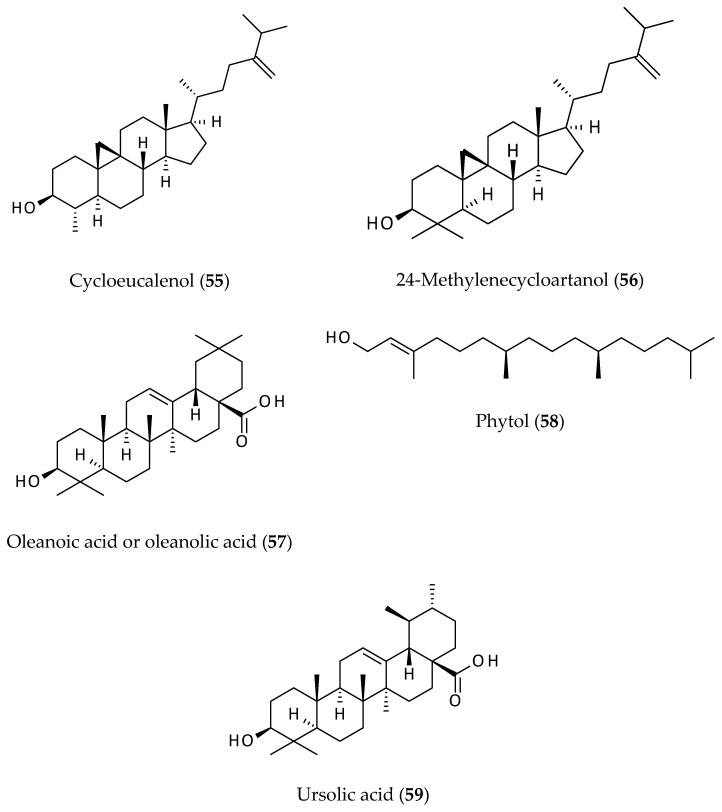
Terpenoids from *Alternanthera philoxeroides*.

Five different terpenoids (**55**–**59**) have been reported from *A. philoxeroides* to date [11,12,14]. Among them, four are triterpenes, cycloeucalenol (**55**), 24-methylenecycloartanol (**56**), oleanoic acid or oleanolic acid (**57**), and ursolic acid (**59**) and one is a diterpene, phytol (**58**) (Figure 9). All of these triterpenes (**55**–**57**, **59**) are well-known for having various biological activities including anticancer, antimicrobial, antiproliferative, and antitumour properties [61,62,63,64].

Triterpenes form a large class of natural products, *ca.* 20,000 discovered to date, with significant differences in structural features and functional groups, and their binding to biomolecules varies considerably. One of the major bioactivities of certain triterpenes is their antiviral property, which are mediated through their interactions with various macromolecules. For example, triterpenes have been shown to manipulate several virus–host fusions by wrapping the heptad repeat-2 (HR2) domain, which are generally found in viral envelops [65], and to inhibit the entry of several viruses (e.g., Ebola, Marbug, human immunodeficiency virus (HIV), and influenza A). Natural triterpenes, particularly cycloartane and oleane classes, were shown to exert their anti-inflammatory, chemopreventive and chemotherapeutic actions through their interactions with various relevant therapeutic macromolecular targets (e.g., cyclooxygenases (COX-1 and 2), lipoxygenase (LOX-5), myeloperoxidase (MPO), phospholipase A2 (PLA2), and inducible nitric oxide synthase (iNOS)) [66].

### 2.9. Miscellaneous

**Figure 10:**
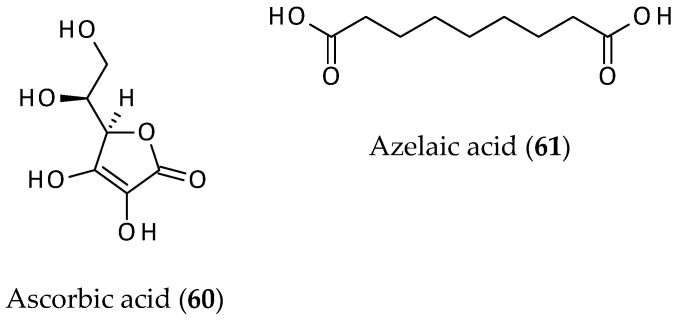
Ascorbic acid and azelaic acid from *Alternanthera philoxeroides*.

In addition to the compounds discussed in the above subsections, there were two other compounds reported from the aerial parts of *A. philoxeroides*, and these are vitamin C (**60**) [67] and azelaic acid (**61**) [12] (Figure 10). Vitamin C (**60**) was not isolated, but its presence in the extract was detected and quantified by a standard chemical assay for ascorbic acid (vitamin C). It was found that the tested sample of *A. philoxeroides* contained *ca.* 34.52 mg/100 g of vitamin C. Vitamin C is an essential vitamin, a well-known antioxidant, and possesses various other beneficial effects, whereas azelaic acid (**61**) is known to have dermatological applications and other pharmacological properties [68,69,70]. Shawon et al. [71] demonstrated molecular recognition (binding affinity and non-bonding interactions) of azelaic acid (**61**) with DNA polymerase I *in silico*.

## 3. Bioactivities and Therapeutic Potential as An Antiviral Agent

Although *A. philoxeroides* is considered as an obnoxious invasive weed around the globe, because of its long-standing traditional medicinal uses, particularly against various viral infectious diseases, this plant and its secondary metabolites have been subjected to various bioactivity studies, revealing the therapeutic potential of this species [2,3,4,5,6,7,8,9,10,11,12,13,16,17,18,29,43,44,72,73,74,75,76,77,78,79,80,81,82,83,84,85,86,87,88,89,90,91], particularly as an antiviral agent (Table 1).

*A. philoxeroides* has long been used in a few traditional medicinal formulations (e.g., Ayurvedic medicines: Swarasa Kalpana, Hima Kalpana and Phanta Kalpana) [72]. Most of the bioactivity assessments performed on this plant and/or its secondary metabolites have been *in vitro* assays, but there have been some *in vivo* animal studies using mice models reported to date. No report on any clinical studies on this plant could be found in the literature. Similarly, only a handful of mechanistic studies providing insights into how certain bioactivities of this plant occur have been published in the literature. Nonetheless, the following subsections present a succinct appraisal of the published literature on the bioactivities, medicinal properties, and therapeutic potential of this plant.

### 3.1. Antibacterial Activity

Antibacterial activity of *A. philoxeroides* has been assessed by various researchers to date (Table 1). However, most of the work has been trivial, and based mainly on the disc diffusion assay. One of the earliest attempts to evaluate the antibacterial activity of the leaves of this plant was made by Rawani et al. [73], where they assessed the aqueous and chloroform–methanol (MeOH) (1:1) extracts of the leaves for antibacterial activity against four bacterial strains, *Bacillus subtilis*, *Escherichia coli*, *Pseudomonas aeruginosa* and *Staphylococcus aureus*, with zones of inhibition of 13.6, 14.1, 17.13, and 13.33 mm, respectively, for the aqueous extract, and 18.27, 14.8, 19.23, and 16.2 mm for the chloroform–MeOH (1:1) extract, respectively. The minimum inhibitory concentration (MIC) values for both extracts against these four strains were also determined and were in the range of 35.25–80.0 μg/mL. Generally, the aqueous extract was less active than the chloroform–MeOH (1:1) extract against all four microorganisms. Preliminary qualitative phytochemical screening revealed the presence of alkaloids, saponins, and sterols, but no flavonoids in the extracts, suggesting that the antimicrobial activity could be attributed to alkaloids, saponins, and sterols, some of which are known to possess antibacterial properties [21,49,56,57,58,59].

Similar antibacterial activity of a MeOH extract of the leaves was also observed against a different *E. coli* strain and *Micrococcus luteus* with zones of inhibition of 52.14 and 34.0 mm at a concentration of 60 μg/mL, and the MIC values of 11.23 and 16.23 μg/mL, respectively [6]. The extract at two other concentrations of 20 and 40 μg/mL also displayed reasonable zones of inhibition of bacterial growth. It was assumed that the antibacterial activity of the extract might be due mainly to the presence of several phenolic compounds (e.g., chlorogenic acid (**31**), ferulic acid (**33**), kaempferol (**21**), salicylic acid (**36**), and syringic acid (**37**)) as certain plant phenolics are known to possess an antibacterial property [74,75]. Interestingly, Kleinowski et al. [7], however, did not find any antibacterial activity of an aqueous ethanol (EtOH) (70%) extract of the leaves against *Bacillus cereus*, *Bacillus subtilis, Escherichia coli, Pseudomonas aeruginosa*, and *Staphylococcus aureus* using the disc diffusion assay, and the organic extracts (e.g., *n*-hexane, dichloromethane (DCM), ethyl acetate (EtOAc), and *n*-butanol extracts) were also totally inactive at test concentrations. This could be due to a variety of reasons, for example, the plant sample might not have been identified correctly (i.e., worked on a wrong sample), different geographical origin, collection time, drying process, and extraction method, just to mention a few.

A bioactive fraction of a MeOH extract incorporated with gold nanoparticles was found to enhance antibacterial potential against *Acinetobacter lwoffii*, *Bacillus subtilis*, *Escherichia coli*, *Micrococcus luteus,* and *Pseudomonas aeruginosa* [76]. In a recent study [17], the potential of a MeOH extract of the aerial parts of this plant against multi-drug resistant (MDR) bacterial strains (a total of 119 clinical isolates) has been explored. It was found that the maximum level of antibacterial activity was against MDR *Staphylococcus saprophyticus*, moderate against MDR *Escherichia coli* and *Proteus vulgaris*, and the least activity was against MDR *Proteus mirabilis,* with the MIC values ranging from 12.5 to 25 μg/mL. In the initial screening using the disc diffusion assay, the extract showed zones of inhibition in the range of 8.33–15.33 mm at a concentration of 750 μg per disc observed against MDR *Enterococcus faecalis*, *Escherichia coli*, *Klebsiella pneumoniae*, *Proteus mirabilis, Proteus vulgaris, Pseudomonas aeruginosa*, *Staphylococcus aureus,* and *Staphylococcus saprophyticus*. The activity profile of the extract was in the following order against: *S. aureus* and *P. vulgaris* > *S. saprophyticus, K. pneumoniae* > *E. faecalis,* and *P. mirabilis* > *P. aeruginosa* and *E. coli* in the disc diffusion assay. Overall, this study was much more comprehensive than any other previous studies on the antibacterial activity of this plant and established antibacterial potency against several MDR clinical isolates of pathogenic bacterial strains. While this finding could be of great interest as MDR bacterial infections (e.g., methicillin resistant *Staphylococcus aureus* (MRSA)) have become one of the major health concerns of the modern world with conventional antibiotics rapidly becoming inactive, prompting the need for new and effective antibacterial agents. The only drawback of this study was that no attempt was made to isolate antibacterial compounds from the active extract. Additionally, any possible modes of the antibacterial actions of the extracts were not investigated. It is crucial that the mechanisms of action of medicinal plant extracts must be understood clearly for optimal utilisation of these extracts as antibacterial agents in phytotherapeutic interventions. Another recent study [18] on the antibacterial activity of this plant was carried out on a MeOH extract of the aerial parts, showing a low level of antibacterial activities, as evident from low zones of inhibition against *Bacillus subtilis* (9.2 mm), *Escherichia coli* (7.7 mm), *Salmonella typhi* (7.3 mm), *Staphylococcus aureus* (6.8 mm), *Staphylococcus epidermidis* (7.3 mm), and *Vibrio cholerae* (5.8 mm) at a dose of 12 mg/disc. The diameter of the disc used in this study was 6 mm.

Akbar et al. [3] evaluated the antibacterial property of the *n*-hexane, chloroform, EtOAc, and MeOH extracts of the leaves, stem, and roots against phytopathogenic bacterial strains, *Erwinia carotovora*, *Ralstonia solanacearum*, and *Xanthomonas axonopodis* and observed the highest level of activity against *Ralstonia solanacearum* (28.1 mm, *n*-hexane extract of the leaves). *n*-Hexane, chloroform, EtOAc, and MeOH leaf extracts produced, respectively, zones of inhibition of 22.0, 20.0, 21, and 19.55 mm against *Erwinia carotovora.* Similarly, the zones of inhibition produced by these four extracts against *Ralstonia solanacearum* were 28.1, 19.08, 26.0, and 21.13 mm, respectively, while 21.5, 16.16, 18.63, and 19.80 mm were against *Xanthomonas axonopodis*. Similar activities were observed with the extracts obtained from the stems and roots. This appears to be the only study on this plant to have looked at the antibacterial activity against phytopathogenic bacterial strains.

### 3.2. Anticancer and Antitumour Activity

The first ever assessment for any potential anticancer/antitumour activity of the crude extract as well as isolated compounds from *A. philoxeroides* was carried out by Fang et al. [5]. Alternanthin (**13**), alternanthin B (**14**), and *N-trans*-feruloyl-3,5-dimethoxytyramine (**2**), *N-trans*-feruloyl-3-methyldopamine (**3**), *N-trans*-feruloyl-tyramine (**4**), and *N-cis*-feruloyl-tyramine (**5**) were isolated from an EtOH (95%) extract of the aerial parts and tested for *in vitro* antitumour activity against *Henrietta Lacks cervical cancer cells* (HeLa) and mouse fibroblast (L929) cells. All compounds showed considerable inhibition of cell growth at a concentration of 30 μg/mL, but at a lower concentration of 10 μg/mL, compounds **2**–**4** and **13** showed little or no inhibition against L929 cells. *N-trans*-feruloyl-tyramine (**4**) was the most active compound among the tested compounds against HeLa cells (72.2% inhibition), whilst alternanthin (**13**) was the most potent against L929 cells (74.9% inhibition) at a concentration of 30 mg/mL. Alternanthin B (**14**) showed a reasonable antitumour activity (50.3% inhibition) against L929 cells, and *N-trans*-feruloyl-3,5-dimethoxytyramine (**2**) was highly active against HeLa cells (72.1% inhibition) at a concentration of 30 μg/mL. It is interesting to note that the only structural difference between alternanthin (**13**) and alternanthin B (**14**) is the presence of a methoxyl functionality in **13**, and a hydroxyl in **14**, and even this minor difference could affect the selectivity and potency of antitumour activity. The least antitumour activity was observed with *N*-*trans*-feruloyl-3-methyldopamine (**3**) against L929 cells with only 13.2% inhibition of cell growth as a concentration of 30 μg/mL.

In continuing the search for anticancer/antitumour compounds from *A. philoxeroides*, seven triterpene saponins, chikusetsusaponin IVa methyl ester (**39**), hederagenin 3-*O*-β-D-glucuronopyranoside-6′-*O*-methyl ester (**41**), oleanolic acid 3-*O*-β-D-glucuronopyranoside (**42**), oleanolic acid 3-*O*-β-D-glucuronopyranoside-6′-O-methyl ester (**43**), oleanolic acid 28-*O*-β-D-glucopyranoside (**45**), and megastigmanes, 4,5-dihydroblumenol (**29**), and 6*S*,7*E*,9*R*-6,9-di-hydroxymegastigma-4,7-dien-3-one-9-*O*-β-D-glucopyranoside (**30**), were isolated from an *n*-butanol extract of the aerial parts of this plant, and the compounds were subjected to screening for antitumour activity against HeLa and L929 cells using the 3-(4,5-dimethylthiazol-2-yl)-2,5-diphenyltetrazolium bromide (MTT) assay [9].

Among the tested compounds, oleanolic acid 3-*O*-β-D-glucuronopyranoside (**42**) was the most active one and displayed 91.3 and 92.9% inhibition of HeLa and L929 cells, respectively. The same group of researchers [8] continued to isolate further triterpene saponins, philoxeroidesides A–D (**47**–**50**), from an EtOH (95%) extract of the aerial parts and assessed their cytotoxicity against human neuroblastoma (SK–N–SH) and human leukaemia (HL60) cell lines. All saponins were cytotoxic to these cell lines with IC_50_ values ranging from 37.29 to 271.45 μg/mL. Philoxeroideside D (**50**) exhibited the highest level of cytotoxicity against SK–N–SH and HL60 cell lines with inhibitory concentration 50% (IC_50_) values of 37.29 and 45.93 μg/mL, respectively. Among these structurally related saponins, compounds (**47**–**49**) showed extremely weak cytotoxicity against HL60 cells with IC_50_ values of 185.29, 185.57, and 271.45 μg/mL, respectively.

In a more recent work, an EtOH extract of the leaves of A. *philoxeroides* was tested against the human osteosarcoma cell line MG-63 and a 67.37% inhibition of cell growth at the concentration of 300 μg/mL was observed [2]. The IC_50_ value was determined as 249.2 μg/mL. While no attempt was made to isolate compounds responsible for this activity, a broad statement was made based on the previously reported phytochemical profile of this plant that the activity might be due to the presence of various bioactive alkaloids, flavonoids, and phenolics [2]. Further investigation is obviously needed to understand how some compounds from A. *philoxeroides* kill certain tumour cells, and it is also essential to establish their toxicities toward human nontumour cells. On a positive note, as some of these studies were conducted with purified compounds with elucidated structures, the findings might prompt rationale drug synthesis based on some of these structures.

### 3.3. Antidementia Activity

Dementia is a generic term to describe a range of progressive malfunctioning of brain cells adversely affecting memory functions, and Alzheimer’s disease appears to be the most common type of dementia. Several plant products (e.g., *Ginkgo* *biloba* extract, ginseng extract, coconut oil, omega-3 fatty acids, curcumin, and resveratrol, and vitamins (e.g., vitamin B_12_ and vitamin D) have long been used for the prevention and treatment of dementia [77]. Khamphukdee et al. [78] recently evaluated an EtOH extract of the whole plant of *A. philoxeroides* for its potential in the treatment of dementia, using various assays including *in vitro* antioxidant assays, β-amyloid aggregation inhibition, and cholinesterase inhibitory activity assays as well as an *in vivo* Morris water maze task, novel object recognition task, and Y-maze task assays. The extract as well as its flavonoids (**13**–**27**) offered inhibition of β-amyloid aggregation. Daily administration of the EtOH extract was found to improve cognitive deficit-like behaviour of ovariectomised mice and to reduce oxidative stress by inhibiting lipid peroxidation in the brain. Based on the experimental findings, it was suggested that *A. philoxeroides* could be considered for the treatment of senile dementia in menopausal and ovariectomised women. The antidementia activity of these flavonoids is mainly associated with their antioxidant and cholinesterase inhibitory properties.

### 3.4. Antidepressant-Like Activity

Khamphukdee et al. [29] studied the potential of A. *philoxeroides* in improving the anxiety-like behaviour of ovariectomised mice using the elevated plus maze, light/dark transition, and locomotor activity tests. It was found that this plant could successfully reduce anxiety-like behaviour in test mice. It was hypothesised that the activity might be due to the presence of flavonoids, particularly, kaempferol (**21**), quercetin (**23**), and rutin (**24**), which are known to possess this activity [79,80]. In a continuation of their study, in the following year, the same group of researchers reported a considerable *in vivo* antidepressant-like activity of an EtOH extract of the aerial parts on ovariectomised mice using the tail suspension and forced swimming tests [11]. It was observed that the crude EtOH extract could ameliorate the depression-like behaviours of the oestrogen-deprived mice. Furthermore, the extract could recover the weight and volume of the uterus of the ovariectomised mice and upregulate the expression of the brain-derived neurotrophic factor (BDNF) mRNA in the hippocampus and frontal cortex, similar to the oestrogen replacement therapy (ERT) drug 17β-oestradiol. In this study, they isolated a series of flavonoids including alternanthin (**13**), alternanthin B (**14**), chrysoeriol 7-O-rhamnoside (**19**), demethyltorosaflavone B (**20**), luteolin 8-C-E-propenoic acid (**25**), and torosaflavone (**27**), and the observed antidepressant-like activity was assumed to be associated, at least partly, with these flavonoids. It was further postulated that this activity might also be a result of the effect of the extract and its phytochemicals on monoamine oxidases (MAOs).

### 3.5. Antihyperglycaemic Activity

Antihyperglycaemic activity of A. *philoxeroides* was first reported about a decade ago [81]. A MeOH extract of the whole plant was evaluated *in vivo* for antihyperglycaemic activity through oral glucose tolerance tests in glucose-loaded mice, and a 65.6% reduction in serum glucose level at a dose of 400 mg/kg body weight was observed. The overall antihyperglycaemic effect was dose-dependent; the inhibitions of serum glucose levels at 50, 100, 200, and 400 mg/kg body weight were 36.3, 58.6, 65.0, and 65.6%, respectively. The effect was comparable to that of the well-known antihyperglycaemic drug glibenclamide, which showed 42.7% inhibition at a dose of 10 mg/kg body weight. This study, however, did not go all the way to isolate compounds responsible for this activity. A couple of years later, Bhattacherjee et al. [6] reported the α-glucosidase inhibitory property of a MeOH extract of this plant, which might be one of the mechanisms by which this plant offers antihyperglycaemic activity. In that study, the IC_50_ value of the extract was determined as 52.41 μg/mL, whereas that of the flavonoid luteolin (**22**) was 36.42 μg/mL. The α-glucosidase inhibitory property was ascribed to various phenolic compounds (e.g., chlorogenic acid (**31**), ferulic acid (**33**), kaempferol (**21**), salicylic acid (**36**), and syringic acid (**37**)) present in the extract. However, as previous phytochemical investigations on this plant revealed the presence of luteolin (**22**) and its glycosides (**17**, **18**, and **25**), it may be assumed that this activity could also be, at least partly, because of the presence of those flavonoids.

### 3.6. Antinociceptive Activity

A MeOH extract of the whole plant of *A. philoxeroides* was assessed *in vivo* for antinociceptive activity by observing attenuation of the number of constrictions in acetic acid-induced gastric pain in Swiss albino mice [81]. A 44.8% reduction in constriction was observed at a dose of 400 mg/kg body weight. The effect was dose-dependent, and at other doses of 50, 100, and 200 mg/kg body weight, the reductions in the number of constrictions were, respectively, 31.0, 32.7, and 37.9%. The finding was comparable to the effect observed with the well-known antinociceptive drug, aspirin, which reduced the number of constrictions by 37.9 and 67.2% at 200 and 400 mg/kg body weight, respectively. Unfortunately, no inference was made to any of the previously reported compounds from this plant, which might be responsible for this activity.

### 3.7. Antioxidant Activity

At least four different reports are available on the antioxidant property of A. *philoxeroides* [6,15,18,78]. The phytochemical profile that we know from the published literature about this species could easily support its antioxidant activity, as this plant is rich in natural antioxidants, ascorbic acid, anthraquinones, flavonoids, and other phenolics [4,5,6,10,11,12,76]. A MeOH extract of the leaves of this plant was found to possess significant antioxidant property in various *in vitro* assays (e.g., 2,2′-azino-bis-3-ethylbenzothiazoline-6-sulphonic acid (ABTS) (IC_50_ = 60.76 μg/mL) and 2,2-diphenyl-1-picrylhydrazyl (DPPH) (IC_50_ = 33.94 μg/mL)) [6]. In the same year, Tukun et al. [15] reported this activity based on the results obtained from the DPPH assay (0.14 μmol Trolox per gram equivalent). Most recently, a more extensive antioxidant activity assessment was performed on a MeOH extract of the aerial parts using a series of *in vitro* assays (e.g., total antioxidant activity (TAA) (3.72 mg AAE/g of extract), ferric reducing antioxidant power (FRAP) (14.73 mM Fe^2+^/mg of extract), DPPH (IC_50_ = 758.55 μg/mL), ABTS (IC_50_ = 586.34 μg/mL) and superoxide (SO) (IC_50_ = 659.7 μg/mL)) [18]. A similar work was carried out on an EtOH extract of the whole plant using the DPPH (IC_50_ = 222.58 μg/mL) and ABTS (IC_50_ = 384.0 μg/mL) assays [78].

### 3.8. Antiviral Activity

One of the major traditional phytotherapeutic uses of A. *philoxeroides* is in the treatment of various viral diseases such as herpes zoster, influenza, and measles, and because of this, a great deal of work has gone into the assessment of the antiviral potential of this plant to date [10,13,43,44,82,83,84,85,86,87,88,89]. The first ever antiviral efficacy testing of this plant was performed on its EtOH extract against the tobacco mosaic virus, not against any human pathogenic virus [82], but the subsequent antiviral studies on various extracts of this plant involved several human disease-causing viruses. Niu [83] reported the antiviral activity of an EtOH extract of the aerial parts against human influenza virus, whilst anti-HIV activity of an aqueous extract was demonstrated by Zhang et al. [84]. An aqueous extract of the aerial parts of *A. philoxeroides* was found to inhibit the growth of HIV *in vitro* at a nontoxic concentration to the host cell with a MIC value of 1.8 mg/mL [84]. At a concentration of 15 mg/mL, the extract was not able to inhibit HIV reverse transcriptase, but with 60 and 120 mg/mL concentrations, it could inhibit by 50 and 95%, respectively. The subtoxic concentration for the extract was found to be 29 mg/mL, at which the extract could inhibit HIV induced cell fusion. It was further observed that the extract could inhibit the growth of Herpes simplex and respiratory syncytial viruses, but not the vesicular stomatitis, adeno, and polio viruses. Based on chemical analyses, it was indicated that the antiviral activity might not be because of phenolic compounds but due to some sulphonated polysaccharides.

A year later, Yang et al. [43] studied various solvent extracts (e.g., petroleum ether, ether, and EtOAc) of several parts of this plant for antiviral activity against the epidemic haemorrhagic fever virus. The petroleum ether, ether, and EtOAc extracts were found to be the active ones. This initial finding was further confirmed by a few more *in vivo* antiviral activity tests against the epidemic haemorrhagic fever virus in suckling mice models [85,86]. Various solvent extracts of the aerial parts were assessed *in vitro* for anti-dengue virus activity and the petroleum extract was found to be the most active (ED_50_ = 47.43 μg/mL), while a microemulsion of the extract was active against coxsackie virus group B-3 [88,89]. *In vivo* antiviral activity of an EtOH extract of the aerial parts was observed against the respiratory syncytial virus in the mice model [87].

One of the most elaborative antiviral screenings on *A. philoxeroides* was performed by Rattanathongkom et al. [13]. Saponins, chikusetsusaponin IVa (**38**) and calenduloside E (**42**), isolated from an EtOH extract of the whole plant, were tested for their antiviral potency. Chikusetsusaponin IVa (**38**) exhibited antiviral activities against Herpes simplex virus (HSV-1 and HSV-2), human cytomegalovirus, measles virus, and mumps virus with selectivity indices (CC_50_/IC_50_) of 29, 30, 73, 25, and 25, respectively, but calenduloside E (**42**) was inactive against all tested viruses. The main difference between these two saponins is that (**42**) contains a carboxylic acid (–COOH) group but in (**38**), this acid functionality is conjugated with a glucupyranosyl moiety, offering more polarity. The mode of action of chikusetsusaponin IVa (**38**) against HSV-2 was studied under different conditions, and it was postulated that the anti-HSV-2 target might be predominantly associated with direct inactivation of virus particles as well as with the inhibition of the release of progeny viruses from infected cells. However, the anti-HSV-2 target was not linked to an inhibitory effect on viral attachment, cell penetration, and viral protein synthesis. This antiviral effect was further substantiated *in vivo* in a mouse model of genital herpes caused by HSV-2.

Several rare C-boivinopyranosyl flavones (**13**–**18**) were isolated from the aerial parts of *A. philoxeroides*, and their antiviral potential was tested against HBV [10]. A significant anti-HBV (hepatitis virus) activity of these compounds was observed. The antiviral efficacy was offered through inhibition of the secretion of HBsAg in HepG2.215 and the IC_50_ values were in the range of 11.39–31.54 μM. Chrysoeriol 6-C-β-boivinopyranosyl-4′-O-β-glucopyranoside (**15**, IC_50_ = 22.2 μM), luteolin 6-C-β-boivinopyranosyl-3′-O-β-glucopyranoside (**17**, IC_50_ = 28.65 μM), and luteolin 6-C-β-boivinopyranosyl-4′-O-β-glucopyranoside (**18**, IC_50_ = 31.54 μM) were found to be the most active antiviral compounds among the tested compounds. All three compounds significantly blocked the secretion of HbsAg in a dose dependent fashion; the inhibitions by the compounds (**15**, **17** and **18**) were, respectively, 74.1, 70.6, and 67.3% at nontoxic concentrations of 127–129 μM.

**Figure 11:**
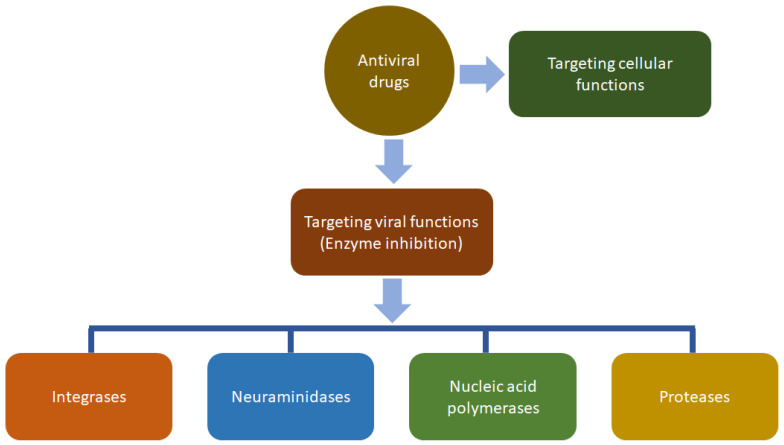
Common modes of action of the currently available antiviral drugs.

The studies conducted to date on antiviral efficacy and plausible modes of action of the extracts of *A. philoxeroides* and compounds isolated from this plant [10,13,43,44,82,83,84,85,86,87,88,89], particularly rare flavonoid glycosides and a few saponins as discussed earlier, certainly support its traditional uses as an antiviral phytotherapeutic option for the treatment of human viral diseases, and reveals their potential as a therapeutic option in modern antiviral therapy. Some of these compounds could also be developed as modern drug formulations, and at least the structural features of these compounds could be exploited to synthesise new antiviral drugs. However, further studies with the extracts as well as isolated compounds from this plant could be extended to gain further insights into whether they directly target the viruses and act as the inhibitors of virus attachment, virus entry, and uncoating as well as inhibitors of various enzymes (e.g., polymerases, proteases, nucleoside, and nucleotide reverse transcriptases, and integrases) (Figure 11). The mechanisms of action of the currently available antiviral drugs, particularly against influenza, could incorporate their transformation to triphosphate following the viral DNA synthesis inhibition and enhancement of the host cells’ resistance to viruses; the mechanisms could also include suppression of the virus adsorption in the cell or its diffusion into the cell and its deproteinisation process in the cells [90].

### 3.9. Cardioprotective Activity

Potential cardioprotective property of a MeOH extract of the leaves of *A. philoxerides* was demonstrated in a study conducted by Zhang et al. [16]. A significant prevention of cardiomyocyte apoptosis induced by doxorubicin using H9c2 cells and determined by the MTT and Annexin V-FITC/PI staining assays was reported. From flow cytometric analysis, it was found that the pretreatment of the extract at concentrations of 10, 20, 40, 80, and 160 mg/mL could decrease H9c2 cell apoptosis induced (~54%) by doxorubicin to 51.18, 42.5, 33.18, 25.2, and 23.46% [16]. It can be mentioned here that H9c2 is a subclone of the original cell line derived from embryonic BD1X rat (Rattus norvegicus) heart tissue. It was suggested that this cardioprotective effect could be because of the β-carboline (**1**) and quercetin (**23**) present in this plant, but possible contributions from other components, particularly, several other flavonoids, have not been ruled out.

### 3.10. Cholinesterase Inhibitory Activity

An EtOH extract of the whole plant of *A. philoxeroides* has recently been evaluated for its cholinesterase inhibitory activity using acetylcholinesterase and butyrylcholinesterase by Ellman’s method [78]. The extract was found to produce an inhibitory effect on acetylcholinesterase and butyrylcholinesterase with IC_50_ values of 2.06 and 3.27 μg/mL, respectively. The selectivity index SI was calculated as a ratio between the IC_50_ values of the inhibition of butyrylcholinesterase and acetylcholinesterase and was found to be 1.60. This activity is likely to be associated with various flavonoids (**13**–**27**) that this plant produces.

### 3.11. Oestrogenic Activity

An EtOH extract of the aerial parts of *A. philoxeroides* displayed *in vitro* oestrogenic activity in the MCF-7 breast cancer cell line [11]. Concentrations of the extract ranging from 1 to 100 μg/mL were used in this study. The extract at a concentration of 1.68 μg/mL showed oestrogenic activity as effective as the effect exerted by 100 pM of 17β oestradiol. Oestrogenic activity of various flavonoids and phenolic compounds are well-known [91], and as this plant produces various flavonoids and phenolics, it is not at all surprising that this extract showed oestrogenic property.

### 3.12. Immunomodulatory Activity

Immunomodulatory activity of the MeOH extract of the aerial parts of *A. philoxeroides*, its n-butanol and ether fractions, and isolated saponin, chikusetsusaponin IVa (**38**), was assessed using the splenocyte proliferation test [13]. The extract and fractions did not show any cytotoxicity to the host cells at a concentration of 200 μg/mL. The ether fraction (50 μg/mL) significantly inhibited splenocyte proliferation, while saponin (**38**) (25 μg/mL) increased splenocyte proliferation in a dose-dependent fashion.

## 4. Toxicological Aspects

While no thorough and systematic toxicological studies have been performed with the extracts of *A. philoxeroides*, a few preliminary studies, for example, an assessment of toxicity towards host cells [84], revealed that the extracts are reasonably nontoxic. Furthermore, as this plant is widely consumed as a leafy vegetable in some countries without the experience of any noticeable toxicity at the amounts consumed, it could be assumed that this plant might not cause any harm to humans. However, looking at the phytochemical profile, one might wonder whether the presence of toxic compounds such as tyramine and β-carboline alkaloids as well as saponins might contribute to certain level of toxicities of the extracts. On a positive note, these compounds are not present in high amounts in the extracts, suggesting that the amounts could be well below the threshold for showing any toxicity in humans.

## 5. Conclusions

Although somewhat neglected as an invasive weed, *A. philoxeroides* has been used as a leafy vegetable and as a traditional medicine for phytotherapeutic interventions in various human ailments, particularly, different viral infections (e.g., influenza and measles). Previous phytochemical studies have furnished the presence of at least 60 different secondary metabolites, where flavonoids and saponins are the two largest phytochemical groups. Various pharmacological and biological activity screenings have unveiled the therapeutic potential of crude extracts as well as isolated compounds from this plant against various diseases, especially, against human pathogenic viruses such as HIV, HBV, and so on. Some of the findings of the reported bioactivities studies certainly provide strong scientific rationale for traditional medicinal uses of this plant, particularly, its uses against viral infections. However, more preclinical and toxicity studies as well as well-designed clinical trials are needed before any confirmed therapeutic recommendations can be made on the crude extracts or purified bioactive compounds from this plant.

## Figures and Tables

**Table 1:** Bioactivities and therapeutic potential of *A. philoxeroides*.

Bioactivities	Brief description	Types of Extract	IC_50_/MIC or/EC_50_	References
Antibacterial activity	Extracts of leaves; considerable activity (zones of inhibition) against *Bacillus subtilis* (13.6 and 18.27 mm), *Escherichia coli* (14.2 and 14.8 mm), *Pseudomonas aeruginosa* (17.13 and 19.23 mm) and *Staphylococcus aureus* (13.33 and 16.2 mm) using the disc diffusion assay.	Water and CHCl_3_–MeOH (1:1)	MIC = 35.25–80.0 μg/mL	[73]
	Extract of the leaves; zones of inhibition against *Escherichia coli* (52.14 mm) and *Micrococcus luteus* (34.0 mm) at a concentration of 60 μg/mL. MIC values were determined against *E. coli* and *M. luteus*.	MeOH	MIC = 11.23–16.23 μg/mL	[6]
	Aqueous EtOH extract (70%) of leaves and stem; no activity against *Bacillus cereus*, *Bacillus subtilis*, *Escherichia coli*, *Pseudomonas aeruginosa* and *Staphylococcus aureus* in disc diffusion assay.	Water–EtOH (3:7)	Not determined	[7]
	Bioactive fraction of a MeOH extract of the aerial parts incorporated gold nanoparticles; improved activity against *Acinetobacter lwoffii*, *Bacillus subtilis*, *Escherichia coli*, *Micrococcus luteus* and *Pseudomonas aeruginosa* using the disc diffusion assay.	MeOH	Not determined	[76]
	Extract of the aerial parts; maximum antibacterial activity against MDR *Staphylococcus saprophyticus*, moderate against MDR *Escherichia coli* and *Proteus vulgaris*, and the least activity against MDR *Proteus mirabilis*.	MeOH	MIC = 12.5–25.0 μg/mL	[17]
	Extracts of the leaves, stem and roots; considerable activity (zone of inhibition) in the disc diffusion assay against phytopathogenic bacterial strains, *Erwinia carotovora*, *Ralstonia solanacearum* and *Xanthomonas axonopodis*, with the highest activity against *Ralstonia solanacearum* (28.1 mm, *n*-hexane extract of the leaves).	*n*-Hexane, CHCl_3_, EtOAc, and MeOH	Not determined	[3]
	Extract of the aerial parts; weak activity (zone of inhibition) in the disc diffusion assay at 12 mg/disc against *Bacillus subtilis* (9.2 mm), *Escherichia coli* (7.7 mm), *Salmonella typhi* (7.3 mm), *Staphylococcus aureus* (6.8 mm), *Staphylococcus epidermidis* (7.3 mm), and *Vibrio cholerae* (5.8 mm).	MeOH	Not determined	[18]
Anticancer and antitumour activity	Alternanthin (**13**), alternanthin B (**14**), and *N-trans*-feruloyl-3,5-dimethoxytyramine (**2**), *N-trans*-feruloyl-3-methyldopamine (**3**), *N-trans*-feruloyl-tyramine (**4**) and *N-cis*-feruloyl-tyramine (**5**), isolated from an EtOH (95%) extract of the aerial parts; *in vitro* antitumour activity of the isolated compounds against HeLA and L929 cells.	EtOH (95%)	13.2–72.2% inhibition at 30 μg/mL	[5]
	Isolated saponins, philoxeroidesides A–D (**47**–**50**) from an EtOH (95%) extract of the aerial parts; cytotoxicity against SK–N–SH and HL60 cell lines.	EtOH (95%)	IC_50_ = 37.29–271.45 μg/mL	[8]
	Isolated compounds from an *n*-butyl extract of the aerial parts; *in vitro* antitumour activity of isolated compounds as determined by the MTT assay, with oleanolic acid 3-*O*-β-D-glucuronopyranoside (**42**) being the most active compound against HeLA and L929 cells at 30 mg/L.	*n*-Butane	91.3–92.9% inhibition at 30 μg/mL	[9]
	Extract of the leaves; *in vitro* cytotoxicity against human osteosarcoma cell line MG-63.	EtOH	67.37% inhibition at 300 μg/mL	[2]
Antidementia activity	Extract of the whole plant; based on *in vitro* antioxidant activity, β-amyloid aggregation inhibition and cholinesterase inhibitory activity, as well as *in vivo* Morris water maze task, novel object recognition task, and Y-maze task. The extract as well as its flavonoids offered inhibition of β-amyloid aggregation.	EtOH	Not determined	[78]
Antidepressant-like activity	Extract of the aerial parts and isolated compounds; considerable *in vivo* antidepressant-like activity on ovariectomized mice using the tail suspension and forced swimming tests.	EtOH	Not determined	[11,29]
Antihyperglycaemic activity	Extract of the whole plant; *in vivo* antihyperglycaemic activity was evaluated through oral glucose tolerance tests in glucose-loaded mice (65.6% reduction in serum glucose level at a dose of 400 mg/kG body weight).	MeOH	Not determined	[81]
	Extract of the leaves; α-glucosidase inhibitory property.	MeOH	IC_50_ = 52.41 μg/mL	[6]
Antinociceptive activity	Extract of the whole plant; *in vivo* antinociceptive activity was evaluated by attenuation of the number of constrictions in acetic acid-induced gastric pain (44.8% reduction in constriction at a dose of 400 mg/kG body weight).	MeOH	Not determined	[81]
Antioxidant activity	Extract of the leaves; active in the ABTS and DPPH assays.	MeOH	ABTS IC_50_ = 60.76 µg/mL and DPPH IC_50_ = 33.94 µg/mL	[6]
	Extract of the aerial parts; DPPH (0.14 µmol Trolox per gram equivalent).	*n*-Hexane-DCM (1:1)	Not determined	[15]
	*In vitro* antioxidant activity of an EtOH extract of the whole plant.	EtOH	DPPH IC_50_ = 222.58 µg/mL and ABTS IC_50_ = 384.0 µg/mL	[78]
	Extract of the aerial parts; TAA (3.72 mg AAE/g of extract), FRAP (14.73 mm Fe^2+^/mg of extract), and active in the, ABTS and SO assays.	MeOH	DPPH IC_50_ = 758.55 µg/mL, ABTS IC_50_ = 586.34 µg/mL and SO IC_50_ = 659.7 µg/mL	[18]
Antiviral activity	Extract of the leaves; antiviral activity against tobacco mosaic virus.	EtOH	Not determined	[82]
	Extract of the aerial parts; antiviral activity against human influenza virus.	EtOH	Not determined	[83]
	Aqueous extract of the aerial parts; antiviral activity against HIV.	Water	MIC = 1.8 mg/mL	[84]
	Various solvent extracts of several parts of this plant; antiviral activity against epidemic haemorrhagic fever virus, with petroleum ether, ether and EtOAc extracts being the active ones.	Petroleum ether, ether, and EtOAc	ED_50_ = 47.43 µg/mL	[43]
	Extract of the whole plant; *in vivo* antiviral activity against the epidemic haemorrhagic fever virus in suckling mice model.	EtOH	Not determined	[85]
	Extract of the aerial parts; *in vivo* antiviral activity against the epidemic haemorrhagic fever virus.	EtOH	Not determined	[86]
	Various solvent extracts of the aerial parts; *in vitro* anti-dengue virus activity, with the petroleum extract being the most active.	Petroleum ether	ED_50_ = 47.43 µg/mL	[44]
	Microemulsion of the extract; *in vitro* antiviral activity against coxsackie virus group B-3.	EtOH	Not determined	[88]
	Extract of the aerial parts; *in vivo* antiviral activity against respiratory syncytial virus in mice model.	EtOH	ED_50_ = 47.43 µg/mL	[87]
	Chikusetsusaponin IVa (**38**) and calenduloside E (**42**), isolated from an EtOH extract of the whole plant; Chikusetsusaponin IVa (**38**) exhibited antiviral activities against HSV-1, HSV-2, human cytomegalovirus, measles virus, and mumps virus, but calenduloside E (**42**) was inactive against all tested viruses.	EtOH	Selectivity indices (CC_50_/IC_50_) = 29, 30, 73, 25 and 25	[13]
	Extract of the leaves; activity against dengue virus.	Petroleum ether	ED_50_ = 47.43 µg/mL	[89]
	Isolated C-boivinopyranosyl flavones (**13**–**18**) from an EtOH extract of the aerial parts; significant anti-HBV (hepatitis virus) activity by inhibiting the secretion of HBsAg in HepG2.215.	EtOH	IC_50_ = 11.39–31.54 µM	[10]
Cardioprotective activity	Extract of the leaves; significant prevention of cardiomyocyte apoptosis induced by doxorubicin using H9c2 cells and determined by the MTT and Annexin V-FITC/PI staining assays.	MeOH	Not determined	[16]
Cholinesterase inhibitory activity	Extract of the whole plant; acetylcholinesterase and butyrylcholinesterase inhibition with IC_50_ values of 2.06 and 3.27 µg/mL, respectively.	EtOH	IC_50_ = 2.06 and 3.27 µg/mL	[78]
Oestrogenic activities	Extract of the aerial parts; *in vitro* estrogenic activity in MCF-7 breast cancer cell line.	EtOH	EC_50_ = 1.68 µg/mL	[11]
Immunomodulatory activity	Extract of the aerial parts, its *n*-butanol and ether fractions, and chikusetsusaponin IVa (**38**); the ether fraction (50 µg/mL) inhibited splenocyte proliferation, but saponin (**38**) (25 µg/mL) increased splenocyte proliferation.	MeOH	Not determined	[13]

## Data Availability

All relevant data have been presented as an integral part of this manuscript.
